# Deployment and validation of a smart bed architecture for untethered patients with wireless biomonitoring stickers

**DOI:** 10.1007/s11517-024-03155-3

**Published:** 2024-07-22

**Authors:** Tânia Nunes, Luís Gaspar, José N. Faria, David Portugal, Telmo Lopes, Pedro Fernandes, Mahmoud Tavakoli

**Affiliations:** Institute of Systems and Robotics, University of Coimbra, Coimbra, Portugal

**Keywords:** Digital healthcare, Internet of Things, Interoperability, Performance metrics

## Abstract

**Abstract:**

Conventional patient monitoring in healthcare has limitations such as delayed identification of deteriorating conditions, disruptions to patient routines, and discomfort due to extensive wiring for bed-bound patients. To address these, we have recently developed an innovative IoT-based healthcare system for real-time wireless patient monitoring. This system includes a flexible epidermal patch that collects vital signs using low power electronics and transmits the data to IoT nodes in hospital beds. The nodes connect to a smart gateway that aggregates the information and interfaces with the hospital information system (HIS), facilitating the exchange of electronic health records (EHR) and enhancing access to patient vital signs for healthcare professionals. Our study validates the proposed smart bed architecture in a clinical setting, assessing its ability to meet healthcare personnel needs, patient comfort, and data transmission reliability. Technical performance assessment involves analyzing key performance indicators for communication across various interfaces, including the wearable device and the smart box, and the link between the gateway and the HIS. Also, a comparative analysis is conducted on data from our architecture and traditional hospital equipment. Usability evaluation involves questionnaires completed by patients and healthcare professionals. Results demonstrate the robustness of the architecture proposed, exhibiting reliable and efficient information flow, while offering significant improvements in patient monitoring over conventional wired methods, including unrestricted mobility and improved comfort to enhance healthcare delivery.

**Graphical abstract:**

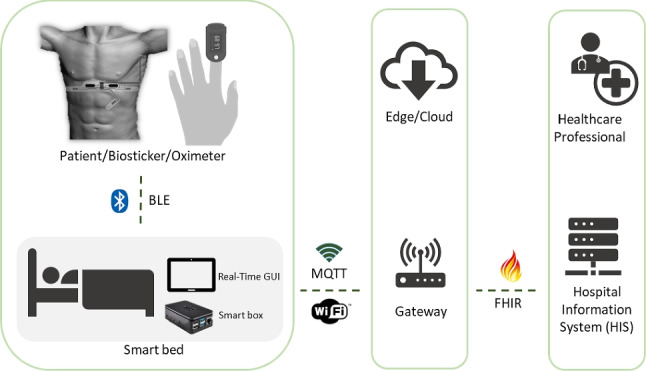

## Introduction

The general increase in life expectancy has led to an accelerated aging of the population and the proliferation of various serious diseases [1]. This is a widespread problem and a major driver for investment in advanced technologies to support the treatment and cure of diseases. Therefore, there is a growing interest in digital health and home healthcare, including miniaturized biomonitoring devices that can wirelessly collect and transmit health data. Unlike consumer-level wearable systems, such as smartwatches, these wearable devices are expected to collect clinical-grade data from various locations on the body. Therefore, the use of smart patches and wearable e-textiles has been the subject of research in recent years [2]. Wireless miniaturized sensors enable greater mobility for hospital patients, while promoting domiciliary hospitalization. However, such solutions differ significantly from current medical devices. Issues such as the placement of the wearable patches, battery charging and replacement, and network communication with other devices should be carefully analyzed in a real-world context to understand the benefits and potential shortcomings of their use. A device with this purpose should be able to seamlessly connect to various networks, securely transmit and record patient vital signs, and relay this information to a central infrastructure to support data management by healthcare professionals, who in turn must receive training on proper and safe use of the system prior to its deployment.

In general, the introduction of new technologies in healthcare must ensure interoperability, i.e., the ability of a device or system to seamlessly integrate and communicate with various other devices to retrieve important data [3]. Within the context of the WoW project,^1^ an innovative architecture that enables wireless biomonitoring of patients together with centralized data acquisition, processing, and transmission was developed [4]. The primary objective is to take a step towards unthethering the patients from their beds in clinical settings and towards domiciliary hospitalization.

**Fig. 1:**
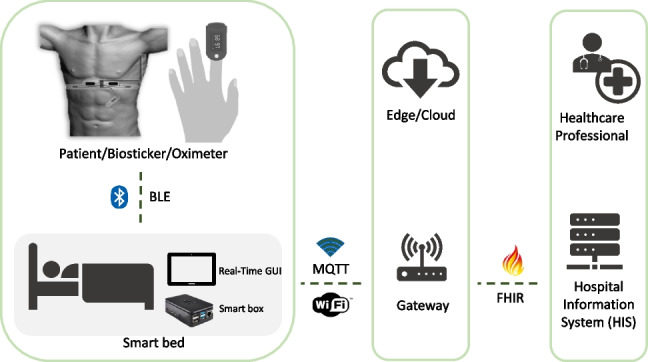
WoW wireless patient biomonitoring system

The devices used to monitor health status have a direct impact on the ability to assess a patient’s condition, safety, prevention, diagnosis, treatment, and rehabilitation. Therefore, it is imperative to test and validate the different system components proposed. The main contribution of this study is to deploy and validate a smart bed architecture for wireless biomonitoring of patients by establishing and evaluating a set of performance metrics. As shown in Fig. 1, the system is organized into three main modules: the smart bed, the gateway, and the hospital information system (HIS). The smart bed retrieves the patient’s vital signs from the wireless biomonitoring devices, namely a biosticker and an oximeter over BLE. The individual patient’s smart box processes the information before transmitting it to the gateway via Wi-Fi. At the same time, this data is organized and displayed in real time on a graphical user interface per patient, allowing for instant visualization. The gateway serves as a central hub that receives data from all smart boxes and converts it into an interoperable format compatible with the HIS. The gateway also manages all connected smart boxes and the data they collect. The HIS acts as front-end software for healthcare providers, allowing them to monitor the vital signs data collected by the wireless smart bed architecture. This gives healthcare professionals a comprehensive overview of the patient’s health status, enabling them to make informed decisions and provide patient care.

Our study focuses on examining the performance of all system modules and their operational efficiency, evaluating the communication between the different components, ensuring user satisfaction with the graphical user interface (GUI), considering all available features and the usability of the system, and analyzing factors such as the ease of setting up and utilizing the system, as well as user comfort.

This paper is organized as follows: Section 2 provides an overview of related work on IoT applications and wearables for monitoring and analysis of patient’s health status through vital signs. Section 3 addresses the methods and materials involved in this study and outlines the performance metrics designed to evaluate the system in real-world scenarios, both from a technical and a usability perspective. In Section 4, we focus on the experimental validation of the hospital pilot setup and discuss the results obtained. Finally, concluding remarks are described in Section 5, and future work is discussed in Section 6.

## Related work

The Internet of Things (IoT) has found diverse applications across numerous domains due to its versatility and potential for transformative impact. Applications include Robotics [5], where IoT enables interconnected systems to enhance automation and efficiency; smart cities [6, 7], where IoT technologies facilitate data-driven urban management, optimizing resources and infrastructure; smart homes [8], empowering residents with remote monitoring and control over domestic systems for improved convenience and energy efficiency; and other application domains such as agriculture, transportation, and industrial automation [9, 10], demonstrating its capacity to revolutionize various aspects of modern life.

Particularly relevant in the context of this work is the integration of IoT into healthcare, which has led the research community to develop new systems to assist healthcare professionals in their daily tasks, from measuring vital signs to preventing diseases such as Alzheimer [11], identifying patient conditions, such as cardio-vascular issues [12] or abnormal beat detection [13], and providing security to the access of personal healthcare information in wireless body area networks [14] and through blockchain technology [15]. The COVID-19 pandemic has reinforced the importance of IoT applications in this field, as the ability to access patient data remotely allows for continuous monitoring of critical symptoms and promotes public safety [16].

Rodriguez-Labra et al. [17] have developed a portable multichannel photoplethysmography (PPG) system that contains multiple nodes of pulse oximeters and allows measurement of various vital signs such as pulse rate, cardiac cycle, oxygen saturation, and blood flow. The main motivation is to monitor cardiovascular factors for the treatment of diseases with abnormal blood flow. This wearable device is attached to the sole of the foot to monitor the vessels and detect changes in blood flow volume, respiration, blood oxygen saturation, blood pressure, and heart rate. In addition to measuring vital signs, it is also capable of computing the effectiveness of localization treatment based on the results of sensor fusion. The device was subjected to a series of breathing exercises to analyze the increase and decrease of oxygen saturation and evaluate the data collected by the sensors. One of the main objectives was to extract cardiovascular metrics. However, a detailed analysis of the collected data is missing.

Silmee is a prototype patch that supports Bluetooth 4.0 (dual mode) and includes four types of sensors (electrocardiogram, pulse, temperature, and acceleration) [18]. The device is able to run different reading modes in real time and non-real time, depending on the application scenario. The patch is applied to the chest and the data recorded by the sensors is sent to a smartphone/tablet via a wireless Bluetooth connection. The reported tests are performed on a male subject, whose pulse and ECG signals are analyzed. Unfortunately, an analysis of the remaining vital data is not disclosed in the tests.

VitalPatch is a disposable wireless biosensor developed by Selvaraj et al. [19] for continuous remote monitoring. It consists of a flexible circuit with coated surface electrodes, such as a sensor that acquires bipolar ECG waveforms, body temperature, and core body motion with a triaxial MEMS accelerometer. It has been tested on 57 subjects who applied the patch to their chest and performed three different types of activities, such as postural changes, metronome breathing at 10–30 brpm, activities of daily living (ADL), and more intense exercises such as walking/running on a treadmill at different intensities [19]. The performance of the VitalPatch in terms of heart and respiratory rate was assessed using the mean absolute error (MAE) and standard deviation. The accuracy of step counting was assessed using the absolute percent error (APE) and the false positive rate (FPR), compared to manual reference counts. The accuracy of posture was compared with manual recording. Due to its reasonable accuracy and the fact that it can be used for several days without interfering in daily tasks, this patch has proven to be clinically acceptable for vital signs and for continuous, unobtrusive patient monitoring.

Following the COVID-19 pandemic, a wearable teleme-dicine solution has been developed in [20] that monitors a range of critical physiological signals, including body temperature, heart rate, blood oxygen saturation, respiratory rate, blood pressure, and cough data. The solution provides an Android-based user interface that displays the physiological parameters measured by the wearable monitoring system and presents alerts with a hierarchical color scheme. In terms of hardware validation, ECG and PP signals, chest movements, cough signals, and body temperature were analyzed. In terms of algorithm validation, the computing platform was validated using data from healthy volunteers as well as publicly available datasets, taking into account heart rate, oxygen saturation, blood pressure, respiratory rate, and lung volume. Although the accuracy of the solution is apparently high, the authors acknowledge that more extensive validation on a larger and diverse group of people, including patients, is needed.

Wital is a real-time vital signs monitoring system based on low-cost and widely available off-the-shelf Wi-Fi devices [21]. The detection signal arises from the deformations of the abdomen and chest caused by breathing and heartbeat, and these deformations can affect the propagation of Wi-Fi signals, recorded by the Wi-Fi Chanel State Information. The system was validated in a daily environment with 10 volunteers, 6 men and 4 women. Each participant underwent a 30-min test in different sleeping positions. Respiration and heart rate were measured with an accelerometer attached to the abdomen and a pulse oximeter attached to the fingertip. The experimental results indicate reasonable accuracy values and reduced error rates.

A wearable medical monitoring system has been developed by the AMON EU Project Consortium [22]. It provides complex monitoring, data analysis, and communication functions in a single wearable device worn on the wrist. The target group for this system is high-risk patients who require continuous monitoring, logging, and analysis of their vital signs. Vital signs monitoring includes pulse, blood oxygen saturation, patient temperature, and physical activity. The main components of this solution are a sensor/analog subsystem, a user interface, a communication subsystem, a digital data processing unit, and a subsystem for power supply and management. In the event of a critical situation, recent data is transmitted to the medical center, so that doctors have a basis for a more accurate diagnosis. This wearable device has undergone medical testing to confirm reliable use without compromising accuracy. The study was conducted on 33 healthy volunteers over a period of 70 minutes, and the data was compared with other devices on the market that meet the CE standards. Blood oxygen saturation, blood pressure (systolic, diastolic, and pulse), and cardiac activity (QT, QRS, and heart) were analyzed during this exercise. Statistical analysis was performed for each sensor using the Bland-Altman method [23]. The AMON device was perceived as comfortable by the majority of the subjects (91%). Only three subjects reported pain in the area where blood pressure was measured.

The 5G smart diabetes system proposed by Chen [24] combines wearables, smartphones, and big data. With the aim of developing a sustainable, cost-effective, and intelligent diabetes diagnosis system with personalized treatment, it has undergone tests and evaluations to verify its feasibility. The data is collected from health records focusing only on diabetes-related information and transferred to a cloud platform via the app interface. Tests were conducted on patients with and without diabetes, and three machine learning methods were used to create models for diabetes diagnosis: decision tree, support vector machine (SVM), and artificial neural network (ANN). The results show that the system can effectively provide patients with personalized diagnoses and treatment recommendations.

Wireless Body Area Networks (WBAN) are gaining ground in the wearable device market. An autonomous WBAN wearable sensor has been proposed by Wu et al. [25], which contains multiple sensor nodes that can be attached to different parts of the body to detect body temperature, heartbeat, and falls. A smartphone application has also been developed to display sensor data and provide information about falls. The system is powered by solar energy and has an autonomy of at least 24 hours. However, the tests conducted focused primarily on the energy source of the device, namely solar energy. The study does not present specific metrics on the data obtained through the monitoring of vital signs. This information gap leaves room for further investigation, particularly on the performance in terms of vital signs data collection and transmission.

An IoT system which uses wearable sensors to monitor patients prone to sarcopenia has been developed in [26]. Sarcopenia is a disease that results in a muscle disorder characterized by a decrease in strength and muscle mass that affects a person’s physical performance. This system tracks muscle mass using EMG, IMU, and a force sensor and sends the data via BLE to an Android app and a web-based server. The app connects to the device, records the parameters, stores and sends the data to the server, and compares it with previous examinations. The benefits include avoiding radiation; however, the device requires the app as a gateway. The test setup effectively demonstrated swift interaction between the device and the associated app. Nevertheless, the evaluation process had its limitations. Aside from conducting multiple replication tests on a single subject, the testing approach focused primarily on functionality. Unfortunately, an analysis of the data exchanged between the devices was lacking. Furthermore, it is important to emphasize that tests need to be conducted with real sarcopenia patients to assess the device’s effectiveness in monitoring the progression of sarcopenia-related diseases. In addition, the system lacks the ability to monitor vital signs in real time, and it can only perform scheduled checks.

Tam et al. [27] developed Healthdot, a wearable for continuous monitoring of heart and respiratory rate using chest accelerometry. In their study, which involved 150 post-operative abdominal oncology patients in a tertiary hospital, Healthdot data was compared with gold standard patient monitoring. Healthdot exhibits occasional high heart rate readings and data quality issues. Nevertheless, it is a suitable choice for general wards, as it effectively increases the frequency of parameter monitoring without compromising clinical performance. Future research should focus on practical wearables for general wards and post-discharge monitoring for early detection of complications.

Also worth mentioning is the comprehensive survey presented in [28], which provides a thorough examination of remote monitoring systems for pandemic patients, particularly focusing on the COVID-19 outbreak. It discusses the significance of innovative technologies such as IoT, blockchain, and machine learning in enabling remote patient monitoring, storage, and analysis of vital health data. Through a scoping literature review, the paper categorizes existing research into three main areas: remote monitoring using IoT, secure data storage/sharing via blockchain, and data processing/analysis using machine learning. It identifies key challenges including the scalability of blockchain platforms, data security, and training requirements. The paper concludes by emphasizing the urgent need for smart remote monitoring systems in the face of pandemics and highlights its contribution in providing insights for future research and development in this critical area.

Previous research has overlooked important factors in the quantitative evaluation of vital signs monitoring systems. While the reliability of data and software has been in focus (e.g., see [29]), communication between device components has been largely neglected. Moreover, identifying and implementing solutions that have been validated in real hospital environments is a major challenge. These arise from the logistical difficulties associated with obtaining test subjects, including obtaining the necessary patient availability and consent documentation. Additionally, the inherent complexity of running a pilot project leads to potential disruption to the routine duties of healthcare professionals and raises concerns about the impact on patient treatment and recovery in hospital. Thus, the work described in this article proposes significant contributions, including the following:

- Introduction of several evaluation metrics for technical assessment of architecture components and inter-component communication
- System usability analysis and user satisfaction through post-study questionnaires
- Comparative assessment against hospital-grade equipment
- Detailed experimental validation of the proposed architecture in a real-world clinical setting with human patients
- Extension of in situ hospitalization towards the preliminary deployment of remote domiciliary patient monitoring

**Fig. 2:**
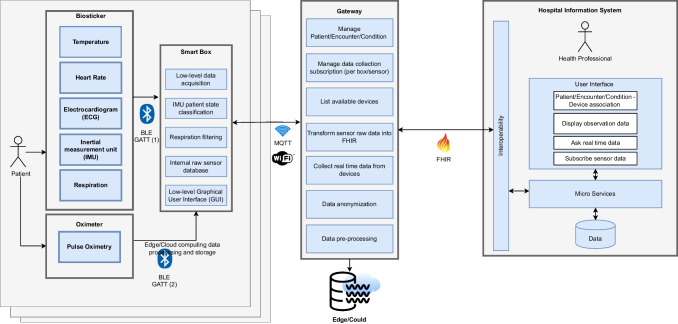
Smart bed architecture for wireless patient monitoring with hospital information system interoperability [3]

## Materials and methods

Performance testing of healthcare equipment plays an important role in ensuring safety, efficacy, and reliability, which benefits patient well-being and promotes healthcare services. Although various systems presented in the literature have been tested in real or controlled environments, the focus of the performance evaluation has been on the accuracy and reliability of the data collected by the sensors. In this assessment, the values recorded by the sensors are often compared to those recorded by existing gold standard measurements commonly used in hospitals. By conducting thorough assessment tests, potential risks and errors can be identified early so that necessary improvements can be made and the potential damage can be minimized.

**Fig. 3:**
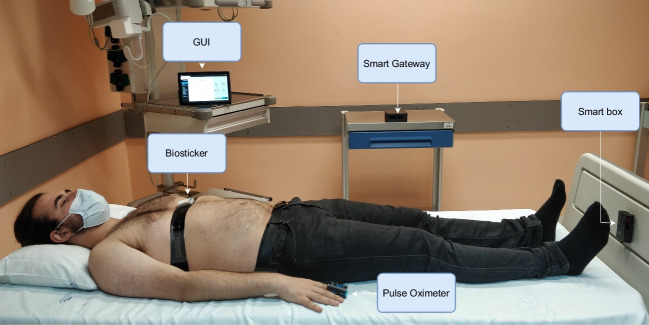
Smart bed architecture patient monitoring in practice [4]

In this work, we devise performance metrics that support test methods for the evaluation of a smart bed architecture for wireless patient monitoring. These are designed to assess and quantify the capabilities of the proposed modules (see below) in terms of their interaction and communication within the system, as well as with external networks. Before delving into these details, it is important to understand how the connection between the devices is established and how they exchange information. The proposed modular and decoupled architecture is illustrated in Fig. 2, which shows all the components and connections between them. In particular, it includes the following elements:

- **Biosticker**: Non-intrusive electronic patches attached to the patient’s skin for acquiring vital signs, fabricated using the technique described in [30]. It contains several sensors, such as electrocardiogram (ECG), heart rate, body temperature, respiratory rate, and an inertial measurement unit (IMU). These signals have been selected on the basis of a preliminary survey with health professionals [31]. A small rigid electronic system is connected to the Biosticker, which transmits the information from the sensors via a Bluetooth Low Energy (BLE) wireless connection.
- **Oximeter**: Commercial instrument that measures the oxygen content in the patient’s blood by attaching it to the patient’s finger. It also transmits the data via BLE.
- **Smart box**: IoT node based on a single-board computer (Raspberry Pi 4B) that collects data from the biosticker and oximeter in real time. Each smart box is assigned to a unique patient and offers a Graphical User Interface (GUI) that displays sensor data in real time and enables the analysis of the various vital parameters of the corresponding patient.
- **Smart gateway**: Based on an Intel NUC8i7BEH, this is the central communication bridge between the smart boxes and the hospital information system (HIS). It manages user data and all smart boxes in the system, including information from connected devices and collected data. It also converts relevant health information into the HL7 FHIR interoperability standard [32] for transmission of electronic health records (EHRs) to the HIS.
- **Hospital Information System (HIS)**: Hospital front-end software that streamlines administrative tasks, patient management, and the control of procedures and processes from the first contact with the patient until they are discharged from the hospital. In our case study, the Globalcare HIS [33] developed by Glintt Healthcare Services has been used. However, the system is compatible with any HIS that can communicate via the FHIR interoperability standard.

Our system prioritizes patient privacy by ensuring that all transmitted data is not linked to a specific individual. Even if the system were breached, only unidentifiable sensor readings would be disclosed. The biostickers are assigned to the smart boxes, which are only linked to the patients by healthcare professionals via the hospital information system (HIS), and not at the IoT system infrastructure level. The architecture includes multiple interacting components that require robust security measures. All data exchanged between the devices and external systems is encrypted so that it is inaccessible to unauthorized personnel. Each device has a Universally Unique Identifier (UUID) for identification and requires authentication. We assign unique cryptographic credentials such as X.509 certificates to each identity and use Transport Layer Security (TLS) and AES-CCM to ensure the confidentiality, authenticity, and integrity of the data. We minimize unnecessary data access, storage, and transmission by implementing role-based access control (RBAC) for MQTT authorization, which restricts a device’s access to its own data. Our system operates in a private network with hidden SSID, MAC ID filtering, and static IP addressing to prevent external interference. We have contingency and mitigation strategies against data breaches, tampering, and DoS attacks. Finally, a data anonymization module in the gateway provides pseudo-anonymization of patient identifiers for additional privacy protection.

Figure 3 demonstrates the practical application of the system, identifying all major components, while portraying a patient laid down in a hospital bed using the proposed wireless monitoring solution.

### Communication between biosticker and smart box

The biosticker and the smart box communicate sensor data using Bluetooth Low Energy (BLE) technology [34], which enables low power consumption and facilitates simultaneous data exchange between the two components.

While there were other suitable candidates such as RFID, NFC, or ZigBee, BLE emerged as the one with the most appropriate characteristics, including data rate, security, and range, without compromising energy efficiency compared to other technologies. The use of a piconet topology enables direct peer-to-peer communication without the need for a central coordinator, as is the case with ZigBee. In addition, BLE supports multiple layers of security that are critical for handling health data through the use of pairing, device authentication, and strong AES-256 encryption, unlike RFID, which also offers a much lower bandwidth. On the other hand, while NFC offers security features, it only has a short communication range of some centimeters, while BLE offers a range of up to 40 meters indoors, which is sufficient for our application scenario. An additional feature of BLE is the use of the Generic Attribute Profile (GATT), which defines how data is organized and exchanged, including support for most sensor types communicated from the biosticker, promoting interoperability.

As the biosticker transmits vital data to the smart box, it becomes essential to ensure the stability of this connection and to check whether any data is lost during testing. If this is the case, the extent of the data loss must be determined. In addition, analyzing the latency of communication between these devices is crucial, as the interpretation of vital signs data by healthcare professionals depends directly on the quality of data exchange. Below, we propose several metrics to evaluate this communication channel.

**Table 1:** Transmission period, transmission rate, and message size for each sensor

Sensor name	Transmission period (ms)	Transmission rate (Hz)	Message size (bytes)
Respiration sensor 1	100	10.0	4
Respiration sensor 2	100	10.0	4
Electrocardiogram	60	16.667	20
Heart rate	5000	0.2	2
Temperature	60,000	0.017	4
Inertial measurement unit	100	10.0	10
Battery	10,000	0.1	1
Oximeter	1000	1.0	4

#### Pairing time

Pairing time refers to the time it takes for the BLE connection to become operational and ready for data transmission, as described by Tosi et al. [35]. In order to measure pairing time, it is assumed that the smart box is ready for data transmission as soon as it receives the pairing confirmation from the biosticker. The pairing time is computed by determining the time interval between the start of scanning for the MAC address of the biosticker by the smart box and the reception of the first record.

#### Number of disconnections

The likelihood that the biosticker will occasionally suffer BLE connection drops, caused by factors such as range or physical obstructions, has also been taken into account. To address this, the smart box logs every disconnection together with a time stamp and a detailed log message.

#### Packet loss

To determine if there are packet losses in the communication between the biosticker and the smart box, we look at the expected amount of data that should have been received by the smart box. This relies on the sampling rate of the sensors embedded in the biosticker. To get a comprehensive insight, Table 1 provides details on the transmission periods and rate, as well as the message sizes for each sensor. This specification allows us to compute the expected data to be received by each sensor and consequently the corresponding packet loss. The total transmission time is determined by using connection, disconnection, or reconnection timestamps from the log messages. Note that, as shown in Table 1, the biosticker has two respiratory sensors, whose data is transmitted to the smart box to calculate the respiration rate, as the biosticker does not perform this calculation directly.

To calculate the expected data to be received and the packet loss for each sensor, the following expressions are used:

1 $$\begin{aligned} \text {Expected data (bytes)} = \left( \frac{\text {Total Transmission Time}}{\text {Sensor Transmission period}} \right) \times \text {Message size}, \end{aligned}$$

2 $$\begin{aligned} \text {Packet Loss (\%)} = \left( 1- \frac{\text {Received data}}{\text { Expected data}}\right) \times 100, \end{aligned}$$

where the received data refers to the size (in bytes) of the specific sensor data actually received and measured by the smart box, during the transmission time.

#### Mean Time Between Failures (MTBF), to repair (MTTR), to fail (MTTF)

To determine the stability of the connection, an indicator can be used that shows how often the connection is interrupted, such as the mean time between failures (MTBF). In addition, the reliability of the connection can be evaluated using the MTTR (mean time to repair), which indicates how long these failures last and how quickly the connection is operational again, and the MTTF (mean time to fail), which indicates how long the connection works on average without interruption.

3 $$\begin{aligned} \text {MTBF} = \text {MTTR} + \text {MTTF}. \end{aligned}$$

#### Throughput

The throughput rate, expressed in bits per second (bps), quantifies the effective data transfer rate during communication [36], which allows us to ascertain the volume of data transferred during the monitoring period for each patient. To assess throughput, we calculate the following:

4 $$\begin{aligned} \text {Throughput per sensor (bps)} = \frac{\text{ Received } \text{ data } \text{ per } \text{ sensor }}{\text {Transmission interval}}, \end{aligned}$$

5 $$\begin{aligned} \text {Total Throughput (bps)} = \sum _{i=1}^{\ \text {N}} \text {Throughput of sensor}\,_i\,. \end{aligned}$$

#### Receive Signal Strength Indicator (RSSI)

The RSSI is a numerical measurement of radio frequency (RF) energy used to assess the suitability of a channel for data transmission [34]. According to the IEEE 802.11 standard, RSSI values range from 0 to −250 dBm, and transmission values between −40 and −103 dBm indicate the readiness of a channel for communication. These values can be retrieved directly via the Bluetooth adapter of the smart box.

**Table 2:** MQTT message size for each sensor

Sensor name	Message size (bytes)
Respiration rate	162
Electrocardiogram	146
Heart rate	150
Temperature	185
Inertial measurement unit	344
Pulse oximetry	155

### Communication between smart boxes and gateway

The connection between the smart boxes and the gateway is established via Wi-Fi, a technology widely used in hospitals and health centers. To ensure reliable communication, Wi-Fi is combined with the Message Queuing Telemetry Transport (MQTT) protocol [37]. MQTT employs a publisher/subscriber approach, in which the sending agent acts as the publisher and the receiving agent as the subscriber. An intermediary, the so-called broker, facilitates communication between the publisher and the recipient.

Communication protocols in IoT are scarce, as the energy-limited devices restrict the use of common, mostly web-based protocols such as Hypertext Transfer Protocol (HTTP). An alternative to MQTT would be Constrained Application Protocol (CoAP). Considering that multiple patients need to be monitored simultaneously, MQTT’s publish/subscribe approach allows for many-to-many communication, while CoAP relies on a request/response approach in a one-to-one communication architecture. Also, while CoAP uses User Datagram Protocol (UDP) for transport, MQTT uses the Transmission Control Protocol (TCP) protocol, which offers higher security via Secure Sockets Layer (SSL)/TLS, which is crucial for healthcare applications. Finally, MQTT offers superior scalability due to its many-to-many architecture while leveraging the lightweight nature of MQTT brokers.

When the sending agent is ready, it initiates a connection request and waits for the broker to confirm the request in order to initiate communication. Besides being lightweight, our implementation benefits from system security through encryption, authentication, and authorization mechanisms [38]. Table 2 depicts the MQTT message sizes of the sensor data sent from the smart boxes to the gateway. Similar to before, this specification allows us to calculate the expected data received for each sensor and consequently the packet loss for MQTT communication. Note that in this case, the respiration data sent to the gateway consists of the respiration rate extracted from the two respiration sensors and not the raw data from the sensors.

#### Packet loss

As previously, to compute the packet loss between a smart box and the gateway, the amount of data sent by a smart box via MQTT and the corresponding data received at the gateway must be known. By comparing these values for each sensor, the packet loss can be determined precisely:

6 $$\begin{aligned} \text {Packet Loss (\%)} = \left( 1- \frac{\text {Received data}}{\text { Expected data}}\right) \times 100. \end{aligned}$$

#### Mean Time Between Failures (MTBF), to repair (MTTR), to fail (MTTF)

To determine the stability of Wi-Fi connections, the MTBF (mean time between failures) incidence metric can be used to determine the frequency of connection failures. To compute this metric, we use the MTTR (mean time to repair) indicator, which provides the downtime of these failures and how quickly the connection became operational again, as well as the MTTF (mean time to fail), which indicates how long the connection works on average without interruption.

7 $$\begin{aligned} \text {MTBF} = \text {MTTR} + \text {MTTF}. \end{aligned}$$

#### Throughput

To compute the throughput between a smart box and the gateway, a similar approach is followed as for the connection between the biosticker and the smart box described in Section 3.1, using Eqs. 4 and 5. This involves assessing the data received at the gateway to determine the communication throughput during the MQTT transmission interval.

It should be noted that the transmission time between the biosticker and smart box within the same set differs from the transmission time between the smart box and the gateway, since these involve different communication mechanisms (via Bluetooth and via Wi-Fi using the MQTT protocol).

#### Round-Trip Time (RTT)

The round-trip time (RTT) is the time elapsed between sending a packet and receiving its acknowledgment [39]. In this work, a smart box sends a sensor message to the gateway and measures the response time. To measure the RTT, the smart box sends a sensor message in a specific MQTT topic every 10 seconds using a rotating queue for each sensor. The gateway responds to the smart box’s request, and the smart box computes the time difference for RTT and logs it for each sensor. The final RTT per sensor is an average of all samples.

### Communication between gateway and HIS

The connection between the gateway and the Hospital Information System (HIS) is established via Wi-Fi. To facilitate seamless data exchange across different systems, the Fast Healthcare Interoperability Resource (FHIR) standard is used [32]. FHIR aims to organize healthcare patterns and enable seamless communication of electronic healthcare data between systems [40].

When it comes to electronic health record (EHR) standards, both OpenEHR and FHIR are widely used. FHIR was chosen for this system because it promotes an architecture that is optimized for data exchange via a REST API while preserving the integrity and heterogeneity of the healthcare devices in use. Overall, it is a more comprehensive standard as it not only defines the format of EHRs, but also provides interoperability rules for data exchange. It also enforces security as the existing REST API uses the OAuth2 authorization protocol.

The evaluated metrics are selected to measure the connectivity performance between the gateway and the HIS, with the aim of ensuring optimal operational efficiency. In contrast to the previous sections, access to the HIS is restricted due to its proprietary closed-source nature. This prevents us from measuring metrics based on a logging system installed in the HIS.

#### Round-Trip Time (RTT)

The RTT can be used to derive the duration between sending a message from the gateway and receiving a response (ACK) from the HIS server. To measure the RTT based on the observations sent to the HIS server, a bundle is created, containing all the necessary information in FHIR standard format. This bundle is then transmitted via an HTTP transaction from a client to an endpoint provided by the HIS. The response acknowledgment (ACK) packet is then captured, enabling the client to compute the total round-trip time. As the messages sent from the gateway to the HIS are HTTP requests, the total time of the request is calculated automatically. Using Java loggers, each time a message is sent for a specific sensor, the RTT is measured and recorded in individual logs per sensor.

#### Throughput

Following the methodology used to evaluate the throughput between the smart boxes and the gateway, as well as the connection between the biosticker-smart box pairs, a similar analysis is performed to evaluate the throughput between the gateway and the Hospital Information System. This metric is determined by considering the capacity of the gateway to transmit information. Since direct access to the HIS is not possible and due to the very low bandwidth required for this communication, we assume in this case that all information transmitted by the gateway is successfully received in the HIS.

### Performance metrics for individual modules

#### Biosticker’s calibration time

After inserting the battery, the biosticker requires a brief initialization phase in which it prepares and calibrates all internal sensors. Following this, the device emits a distinct light signal, indicating that it is now ready to read and transmit data. In this study, the calibration setup time was analyzed to check how quickly the biosticker is ready for communication.

#### Biosticker’s battery

We also analyze whether the battery of the biosticker meets a predefined requirement of at least 24 hours of continuous operation. For this purpose, the battery status is recorded over time, enabling us to determine whether the battery is discharging excessively or functioning as expected.

#### Smart box power consumption

Similarly, the power consumption of the smart boxes is analyzed to understand the power requirements. A USB voltmeter has been used to provide continuous real-time measurements of the smart box’s power consumption. These values are recorded for each different smart box with samples collected within 10-min periods, subsequently subjected to detailed analysis.

**Fig. 4:**
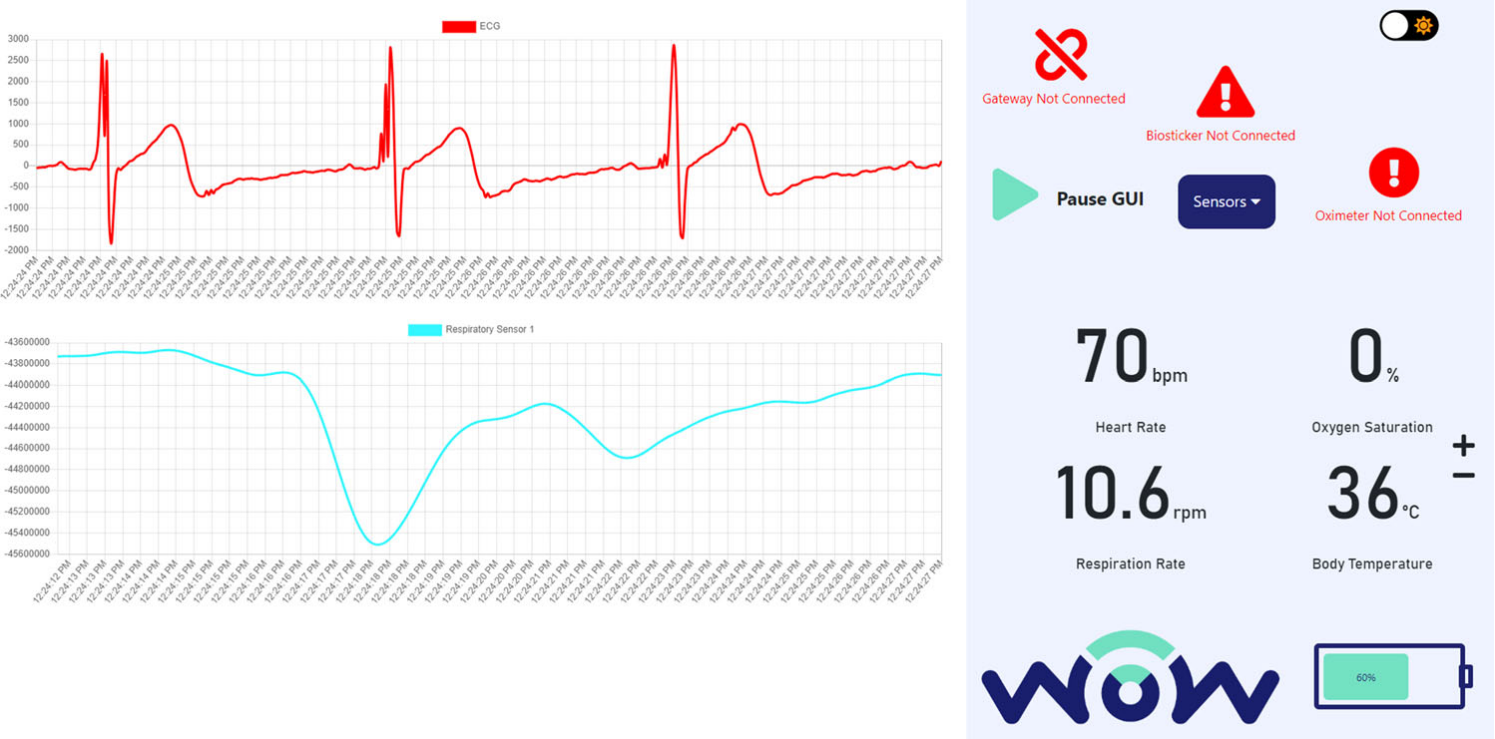
Main menu of the designed user interface. Video available at https://youtu.be/zW7DLGrwWgE

#### GUI testing

Extensive testing ensures a responsive and functional graphical interface to provide an intuitive user experience. The evaluation based on usability principles [41] includes reviewing menus, buttons, and icons to identify potential errors and opportunities for improvement [42]. Evaluating the proposed GUI for displaying each patient’s vital signs is crucial to ensure the interface matches expectations and functions seamlessly. Figure 4 illustrates the main menu of the user interface for a specific patient biosticker. The left side of the screen displays graphical representations of ECG and respiration signals over time, and the right side displays real-time numerical data on heart rate, oxygen saturation, respiration rate, and body temperature. In addition, users can access current device status information, including battery level, connected devices, and visualization modes (e.g., light and dark mode). The technical evaluation focuses on the following:

- Efficient menu navigation with quick transitions and intuitive access buttons
- Effectiveness of dark and light modes
- Placement of error message without obstructing the data view and clear differentiation of error messages and data
- Alignment of graphs and positioning of information for clear readability and interpretation to enable seamless analysis
- Low battery warning messages on the biosticker and prompt notifications (e.g., on disconnections) to enhance user awareness

#### Computational cost of system services

In order to achieve stable and fast operation of the system, the processing software and services running on both the smart box, for individual patients and in the gateway for all patients must be optimized and efficient, and place as little load as possible on the hardware used. To this end, the computational cost of the following services is measured:

- Computational cost of the GUI, measured as a percentage (%) of RAM and CPU utilization on the back-end host (smart box) and on the client front-end (computer/tablet)
- Performance of the local smart box database, measured in terms of query times during operation
- Computational cost of the various services in the gateway (database, MQTT broker, FHIR server, data processing service), measured as a percentage (%) of RAM and CPU utilization

### System usability and user satisfaction questionnaires

Usability testing evaluates the system from the user’s perspective to identify potential problems, such as confusing workflows or unclear instructions [43]. Two user satisfaction questionnaires^2^ were conducted to gain insights. One survey collected feedback from participants who have used the biosticker and focused on their experience, comfort, and views on the proposed system. The second survey was aimed at healthcare professionals, seeking insights into the reliability of the system, data quality, the training and deployment process, the autonomy of the biosticker, design of the GUI, and general views.

### Comparison against gold standard measurements

In addition to the previously presented analytical dimensions, for the experimental validation of the system, we also evaluate the accuracy of the proposed solution with standard instruments used daily in a hospital setting. In particular, we extract the deviation of measurements between instruments to assess the differences between the readings obtained with the WoW biostickers and the gold standard hospital instruments.

**Fig. 5:**
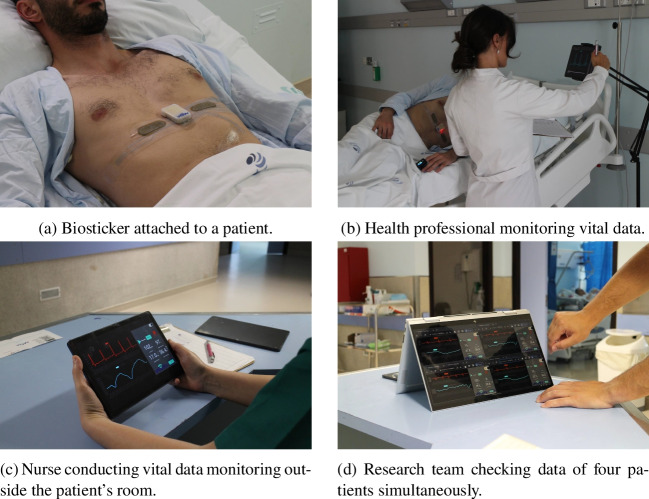
Experimental conditions

**Fig. 6:**
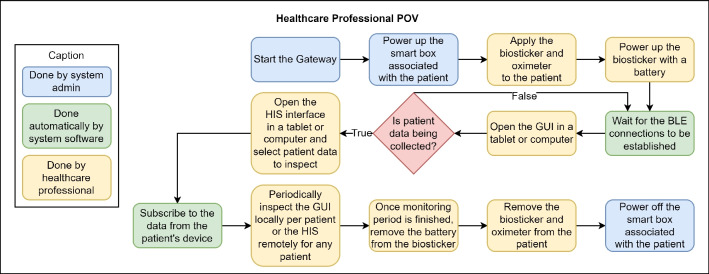
Sequence of actions from the standpoint of healthcare professionals when interacting with the proposed smart bed system

## Results and discussion

In this section, we carry out the experimental validation of the system. We start by presenting the experimental design and describe the experimental setup and the objectives of our experiments. Afterwards, we analyze the different interactions of the system modules as well as their individual performance based on the previously proposed metrics extracted during the pilot experiment. Subsequently, we examine the usability of the system and the satisfaction of the users, taking into account the feedback from the pilot questionnaires. Finally, a comparison of accuracy between the WoW sensors and the hospital gold standard measurements is performed.

### Experimental design and pilot setup

To validate the proposed smart bed architecture for untethered patients with wireless biomonitoring stickers, the system underwent a 3-day pilot experiment in a hospital environment with real patients, who were equipped with a biosticker during their hospital treatment. The pilot took place on three different dates, and a total of nine patients were recruited from the Orthopedic unit of the Hospital and University Center of Coimbra (CHUC), including both genders and distinct reasons for hospital admission. All participants signed an informed consent form to take part in the pilot study.

**Fig. 7:**
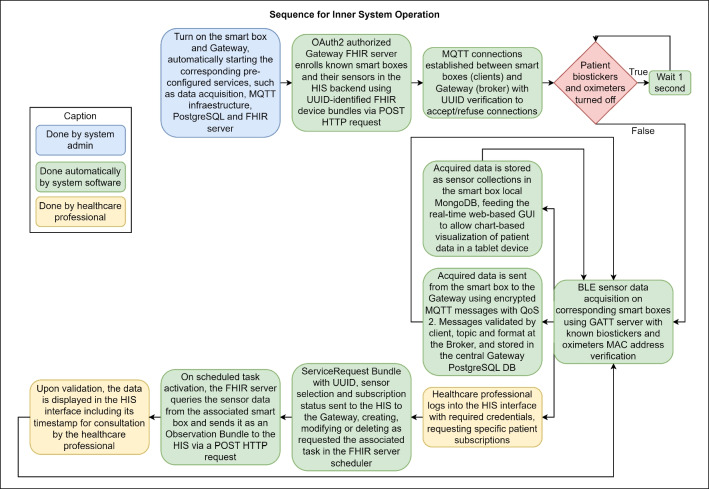
Technical flowchart representing the sequence of steps that materialize the proposed smart bed system

A nurse, who had received prior training with the WoW system, attached the biosticker to the participants. Each patient room contained a smart box and a tablet on a stand that displayed the collected data on the WoW’s GUI, placed near the hospital’s standard instruments for reading vital signs. The research team operated from a dedicated support room, and at least one member of the team remained in the unit for the entire duration of the pilot to ensure continuous monitoring of patient vital signs, recording, and technical support in the event of any issues. Figure 5 presents evidence captured during the pilot conducted at the hospital, including a biosticker attached to a patient, as well as healthcare professionals and the research team reviewing patient vital signs using a tablet.

The healthcare professionals involved were familiarized with the system prior to the start of the pilot based on their previous training. The flowchart in Fig. 6 illustrates in detail the sequence of actions to start and stop the system from the healthcare professional’s perspective, color-coded according to who performs each action. As expected, most tasks are performed by healthcare professionals, who are the main target group of the proposed smart bed architecture.

The research team that deploys the framework has a deeper knowledge of the architecture components and how they communicate with each other. In Fig. 7, which is also presented in the form of a flowchart, we provide a sequential overview of the technical steps for operating the smart bed system, color-coded according to who performs each task. This includes a deeper level of technical detail, particularly on the inner communication mechanisms of the system, such as the FHIR bundles sent over HTTP, MQTT authentication, and the permissions for each client, among others.

**Table 3:** Information of participants involved in the pilot study

Participants	Gender	Age (y)	Admission reason	Monitoringtime (hours)	Mobility level	Smart box ID
Patient A	Female	79	Right humerus fracture; immobilization of the superior right arm	$\approx$ 5 h	Moderate	S1
Patient B	Female	88	Femur fracture post-surgery	$\approx$ 6 h	Moderate	S2
Patient C	Female	84	Right hip prosthesis revision	$\approx$ 24 h	Low	S3
Patient D	Male	59	Right inferior member amputation	$\approx$ 24 h	High	S2
Patient E	Male	77	Left hip prosthesis infection	$\approx$ 5 h	Moderate	S1
Patient F	Male	63	Right knee infection	$\approx$ 6 h	Moderate	S2
Patient G	Male	90	Right transtrochanteric fracture	$\approx$ 22 h	Moderate	S1
Patient H	Female	85	Mechanical failure of osteosynthesis of the left distal femur	$\approx$ 22 h	Low	S2
Patient I	Female	77	Supra-condylar fracture of the left femur	$\approx$ 24 h	Moderate	S3
Remote volunteer	Male	23	Healthy subject	$\approx$ 8 h	Low	S4

Table 3 provides detailed information about the ten subjects that participated in the pilot, such as age, gender, reason for hospitalization, and mobility level. Low mobility refers to patients who are confined to their beds for the entire monitoring period due to their health condition. Hygienic tasks are performed while on the bed. Moderate mobility refers to patients who are hospitalized and may perform assisted sanitary ambulation. High mobility includes patients who are not only able to perform the same mobility activities as the previous groups, but are also able to walk voluntarily within the unit without requiring medical assistance.

The selected patients had similar reasons for admission, such as femur fracture, hip prosthesis anomaly, knee infection, or humerus fracture. Although they had similar diseases, their mobility conditions were different. Patients C and H were restricted in their mobility and confined to the bed except for essential activities. Patients A, B, E, F, G, and I had moderate mobility, which included changing position in bed, chair transfers, and assisted ambulation. Patient D had a high level of mobility and was able to perform all the above-mentioned activities, and also walk voluntarily. In addition to the hospital participants, a remote healthy young male volunteer without mobility impairments was also recruited. The subject was monitored from his home for 8 hours during bedtime using the WoW system. For this reason, we classify his mobility level as low, although the volunteer also used the restroom for his essential needs. This allowed for a fully remote test to examine conditions beyond the hospital setting, representing a first step towards evaluating the system performance in home environments.

Prior to the beginning of the pilot test, the WoW system components were connected to the private hospital network. Each patient received a kit comprising a biosticker, a smart box, an oximeter, and a tablet, as well as a standard hospital monitoring system (Multi-parameter Patient Monitor Mindray iMEC15). Patients were connected to both systems simultaneously, with a nurse in charge overseeing vital signs monitoring in real time, including respiratory rate (manual count), heart rate, ECG, temperature, and SpO_2_ levels.

Questionnaires were given to the participants at the end of the patient observation. As mentioned in Section 3.5, the patients who participated in the pilot study answered a specific questionnaire, while healthcare professionals who were involved in the application of the biosticker or assisted with its application at the beginning of the monitoring period received another questionnaire. The main objectives of this pilot evaluation, based on the metrics presented in Section 3, are as follows:

- Ensure reliable and efficient communication between WoW components.
- Analyze the impact of mobility conditions on device communication.
- Analyze the reliability of communications in both hospital and home environments.
- Ensure that technical factors align with the specifications, such as the battery of the biosticker, the power consumption of the smart box, the initialization of the biosticker, etc.
- Evaluate the usability of the WoW solution and its graphical user interface.
- Obtain feedback from healthcare professionals regarding the sensors integrated in the biosticker and the preference between WoW and existing equipment.
- Gather feedback from patients on their experience with the untethered biosticker.
- Verify the sensor data and compare it with the hospital’s gold standard monitoring measurements.

These objectives allow to assess the performance, usability, and effectiveness of the WoW solution and contribute to its successful deployment for patient biomonitoring, using wireless sensor patches.

**Fig. 8:**
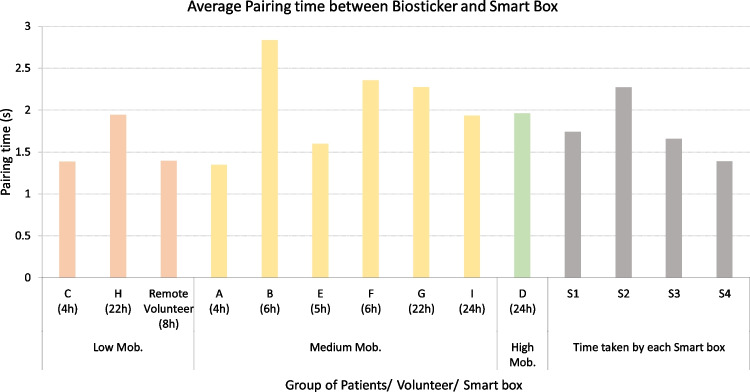
Average pairing time between biostickers and smart boxes

**Fig. 9:**
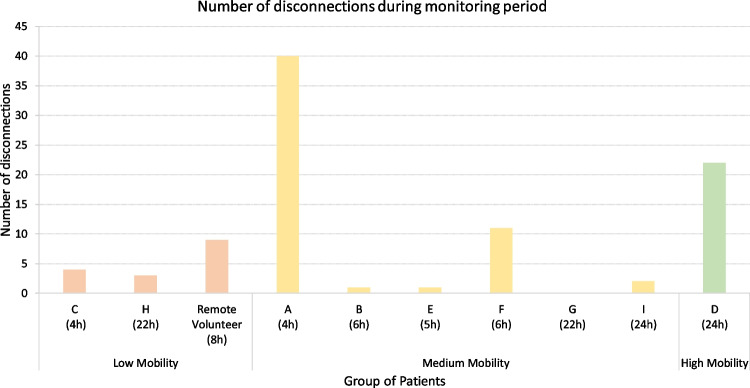
Disconnection analysis along the monitoring period

**Fig. 10:**
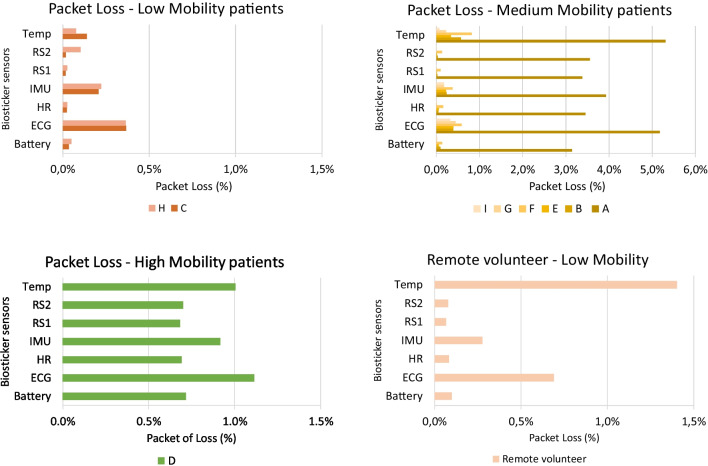
Packet loss (%) between biosticker and smart box by patient group

**Fig. 11:**
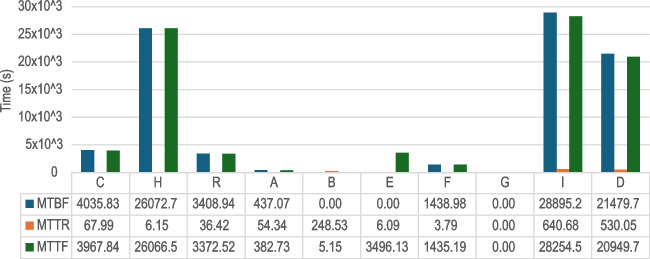
Incident metrics (mean time between failures, to repair, to fail—MTBF, MTTR, MTTF, respectively) for the biosticker-smart box connection

**Fig. 12:**
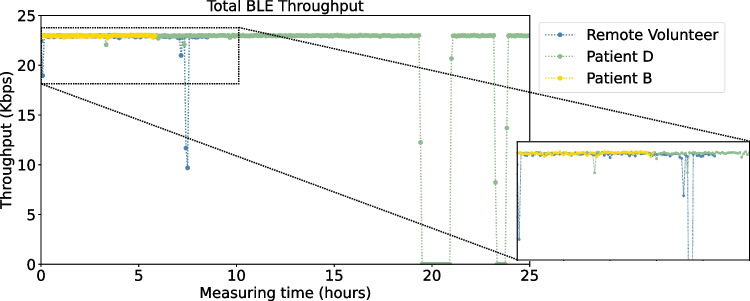
BLE throughput for patient B, D, and remote volunteer

**Fig. 13:**
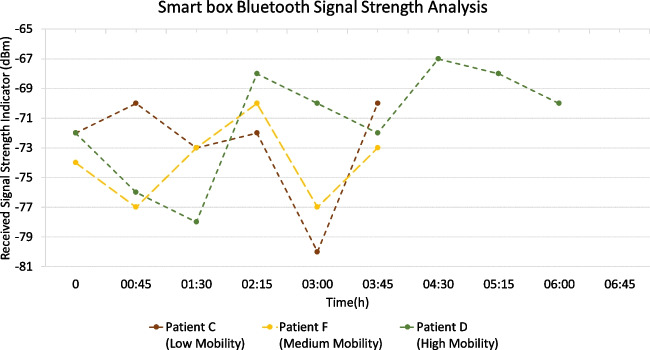
BLE RSSI for patients C, F, and D

It is important to note that pulse oximetry was excluded from most of our analysis below. The commercially available equipment in use proved to be unstable during the pilot, which interfered with data acquisition from its sensor due to inconsistent signal strength. Furthermore, some patients declined to use the oximeter during long-term monitoring, citing discomfort. As a result, the readings were not available.

### BLE communication between biosticker and smart box

To determine the **pairing time** between the biosticker and smart box, an initialization timestamp was recorded, indicating when the smart box began searching for the biosticker to establish a connection. The average pairing time is then computed based on the detection of a connection message in the capture logs.

Figure 8 displays average smart box pairing times, ranging from approximately 1.5 to 2.5 seconds. Patients are categorized into groups according to their mobility status, which are visually distinguished by their color: patients with low mobility are shown in soft red, patients with medium mobility in yellow, and patients with high mobility in green. Additionally, the average pairing time for each smart box is displayed in gray. The color-coded categorization of patients based on their mobility profiles is consistently applied in the following charts.

Patient B had the longest setup, but at 2.88 seconds, it was still within a highly acceptable range. This observation suggests that the system has achieved efficiency in minimizing the time it takes to initiate its operations. The average initiation time per smart box is also shown on the right-hand side of Fig. 8. Notably, smart box 2 exhibits a slightly longer time, possibly due to hardware factors, but it does not significantly affect the initialization process.

While monitoring patients in the hospital setting, we analyzed whether **disconnections** occurred. These disconnections could be caused by various factors, such as improper insertion of the battery of the biostickers, sudden movements of the patient, physical blocking of the biosticker, or the patient leaving the range of the BLE connection to the smart box.

Figure 9 depicts the disconnection counts for each patient/volunteer. Once again, patients are categorized within groups for clarity. When analyzing the chart, it becomes clear that the number of disconnections was significantly higher in two patients than in the others, namely patients A and D. Patient A, who had 40 disconnections, faced this issue primarily due to a fracture in their right arm, which led to an intermittent physical blocking of the biosticker and contact with the battery. Patient D experienced some disconnections due to frequent repositioning and high mobility, causing the biosticker to frequently move out of the BLE communication range of the smart box. In the remote scenario, only minimal variation was observed compared to other patients monitored in the hospital environment.

Throughout the pilot, we also recorded and analyzed the occurrence of **packet loss** between each smart box-biosticker pair. Figure 10 illustrates the packet loss analysis for each patient group, categorized by their respective mobility conditions. For all subfigures, the vertical axes describe the sensors analyzed, while the horizontal axes denote the percentage of packet loss. When examining packet loss, it becomes clear that patient A, experienced a particularly high percentage of packet loss. This phenomenon is due to increased disconnections, which leads to momentary interruptions and, in turn, leads to higher packet loss. Overall, despite the different transmission times and sensor types, the percentage of packet loss remains low, ranging from 0.002 to 5.306%.

The hospital and remote environments showed a similar scenario. Both had higher packet loss rates for ECG and temperature sensors. In the case of ECG, greater data sharing increases the likelihood of loss compared to other sensors. Conversely, the temperature sensor, where little data is shared, results in a more pronounced percentage of packet loss if a value fails. However, the results remained consistent, with the temperature sensor having the highest percentage of packet loss at 1.387%.

We have used the metric **Mean Time Between Failures** (MTBF) to measure the reliability of the connection between biostickers and smart boxes. A direct correlation of MTBF values with patient monitoring time is evident in the study. As shown in Fig. 11, patients with longer monitoring duration have higher MTBF values, with the exception of patient G. Remarkably, despite the longer monitoring duration of this patient, no interruptions in the connection between biostickers and smart boxes occurred, so no failures were observed. For all patients, the MTBF is high as desired (about 1 hour or more), with the exception of patients A and F, who experienced the most disconnections. For the 24-h patients, the MTBF is around the 6-h mark, and for all patients during this study, the MTTR was extremely low, meaning that disconnection times were generally short, demonstrating the resilience of BLE communication.

**Table 4:** Overall BLE throughput performance between biosticker and smart box

	Throughput (Kbps)			
Participants	Mean	Median	Std Dev	Maximum
Patient A	19.41	22.57	6.96	23.00
Patient B	22.96	22.97	0.05	23.04
Patient C	22.33	22.96	3.09	23.03
Patient D	21.08	22.96	6.18	23.03
Patient E	22.05	22.96	4.19	23.03
Patient F	22.80	22.94	0.38	23.03
Patient G	22.95	22.96	0.06	23.04
Patient H	22.95	22.97	0.14	23.02
Patient I	22.46	22.97	3.30	23.04
Remote volunteer	22.62	22.93	1.75	23.00

We have analyzed the **throughput** between biostickers and smart boxes by taking samples every 5 minutes during patient’s monitoring time. Figure 12 visually depicts the results obtained for the throughput (in Kbps), along the patient/volunteer monitoring hours. We focus on three distinct cases: patient B (6 hours), patient D (24 hours), and the remote volunteer (8 hours), whereas Table 4 provides additional details on the throughput outcomes for all participants. We confirm the consistent stability of the BLE throughput analysis on both patients and the volunteer subject, with the values obtained falling within the same range (19–23 Kbps), as shown in Fig. 12. Table 4 presents statistical insights into BLE communication, focusing on key metrics such as mean, median, standard deviation, and maximum throughput. A consistent pattern is discernible across all patients, yet it is notable that patient A exhibits the lowest mean throughput score. This is due to the more frequent and longer disconnections, as previously mentioned. Prolonged disconnections lead to increased packet loss and decreased data exchange between devices.

**Fig. 14:**
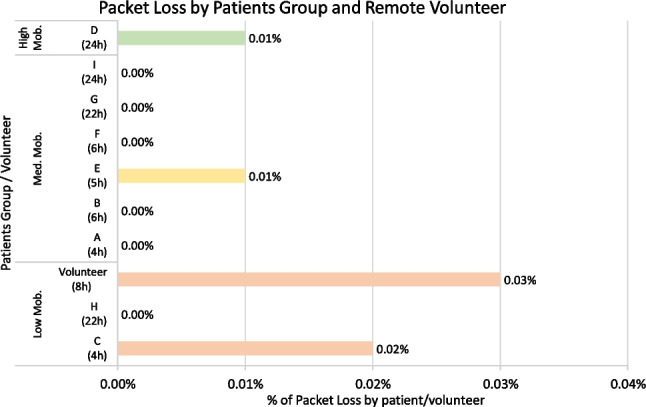
Packet loss for the Wi-Fi communication using the MQTT protocol between smart boxes and the gateway, captured by participant

**Fig. 15:**
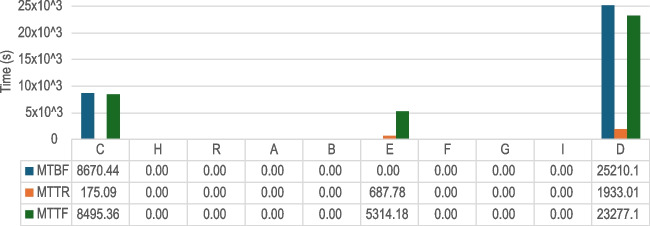
Incident metrics (mean time between failures, to repair, to fail—MTBF, MTTR, MTTF, respectively) for the smart boxes-gateway connection

To assess the communication strength of the biosticker and smart box channels for data transmission, we performed an analysis of the **RSSI** readings. The smart boxes were positioned in close proximity to the patients, so that the distances between each biosticker and the corresponding smart box ranged between 0.8 and 1.5 meters. We measured RSSI at regular intervals every 45 minutes from the start of recording, selecting one patient from each mobility group. These results are shown in Fig. 13. It is worth noting that the RSSI reading time was carefully selected to minimize disruption to the hospital patients’ treatment and daily activities. We selected suitable time periods that were as little disruptive as possible to the patient under analysis.

Based on the RSSI values obtained, it can be seen that the different smart boxes show a consistent and reliable variation between samples. The lowest RSSI value recorded is −80 dBm. Despite this, the quality of the connection remains unaffected as it is still within the range of reasonable RSSI communication values, which are well above the aforementioned reference value of −103 dBM.

**Fig. 16:**
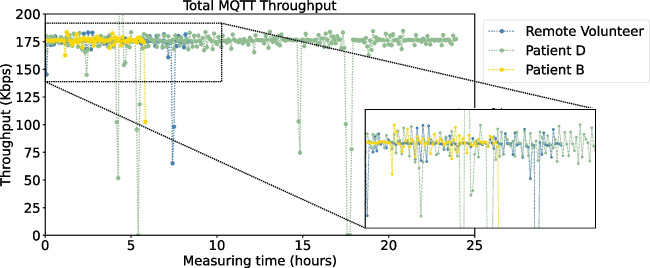
Throughput between smart boxes and the gateway for patients B, D, and remote volunteer

### Wi-Fi communication between smart boxes and gateway

The data received by the central gateway from all smart boxes was analyzed to determine whether packet loss had occurred. During this communication, the smart boxes do not transmit information about their internal battery. Moreover, they compute the patient’s respiratory rate of the patient raw values of the two respiratory sensors. The patient’s respiratory rate is then sent by the associated smart box to the gateway. The remaining sensor data (ECG, heart rate, temperature, IMU and oximetry) is simply relayed to the gateway by all smart boxes.

Figure 14 illustrates **packet loss** between the smart boxes and the gateway for each patient. While three patients (C, D, and E) experienced minor losses resulting in transient data loss, the overall percentage of packet loss remained consistently low, ranging from 0.00% to 0.02%. These can be attributed to momentary drops and instability in the hospital’s Wi-Fi network, leading to sporadic packet loss during transmissions. The results indicate reliable communication between the smart boxes and the gateway, even with longer transmission periods of up to 24 hours. The ability of the system to maintain steady throughput over an extended period of time is guaranteed, as long as the network remains reliable. Factors that contribute to stable communication include positioning the smart boxes in close proximity to the access points (within 10 meters) to ensure appropriate Wi-Fi signal strength. The hospital network used was a stable private network, with minimal load fluctuations, except during peak times, similar to the stability often seen in home networks. In addition, the lightweight MQTT protocol proved to be very efficient, as virtually no packets were lost even during network load peaks.

In the remote environment, the frequency of packet loss is slightly higher than for hospitalized patients. This is somehow expected since the Wi-Fi connection is similar, but it involves communication with the gateway, which in this case is on an external network.

Similar to the analysis of the mean time between failures performed in Section 4.2, a parallel methodology was used to evaluate the communication between the smart box and the gateway. This is illustrated in Fig. 15, which shows extremely stable behavior compared to the BLE counterpart, as expected given the detrimental packet loss observed in Wi-Fi communication between smart boxes and gateway. The vast majority of smart boxes had no connection drops to the MQTT broker, which can be attributed to the stable Wi-Fi link observed in the pilot. The limited number of disconnections was due to unexpected internet availability issues at the facility itself rather than the stability of this communication protocol.

We have extended our investigation to evaluate the **throughput** of communication between the smart boxes and the gateway. The representation of patients across different mobility profile groups was maintained. The pattern of MQTT throughput is similar to that of BLE throughput, where Bluetooth disconnections affect MQTT communication behavior. This is because, when a BLE connection is lost, the data is temporarily unavailable for transmission to the gateway. As soon as the BLE connection is re-established, the data is immediately sent to the gateway. This phenomenon is showcased in Fig. 16, especially in the intervals of 4–10 and 15–20 hours, revealing a gap in the throughput values for the remote subject, patients D and B depicted by the blue, green and yellow data points, respectively. In some cases, when the network was overloaded, MQTT messages queued up before reaching the gateway, causing a decrease in throughput, which was restored when the network was decongested, preventing any packet loss.

Table 5 details the statistical analysis of the MQTT throughput (mean, median, standard deviation, and maximum value) for each participant. As anticipated from the discussion in Section 4.2, patient A exhibits a lower MQTT throughput value. It could be expected that patients D, F, and the remote volunteer would also have lower throughput values than other patients with fewer disconnections. However, while patients C, E, G, H, and I did not register a higher number of disconnections compared to patients A, D, and F, the disconnections were longer, resulting in a lower amount of data exchanged. In contrast, patient B experienced fewer short disconnections, resulting in higher throughput, and a greater amount of successfully exchanged data.

**Table 5:** Throughput performance in Wi-Fi MQTT communication between smart boxes and gateway

	Throughput (Kbps)			
Participants	Mean	Median	Std Dev	Maximum
Patient A	146.61	171.32	53.76	185.52
Patient B	174.98	176.07	9.17	183.45
Patient C	162.06	176.59	48.49	270.45
Patient D	171.37	176.28	26.13	254.04
Patient E	169.18	176.23	32.14	183.19
Patient F	174.87	175.69	3.87	182.96
Patient G	164.70	164.00	10.77	188.69
Patient H	165.90	165.58	11.18	229.69
Patient I	166.96	164.87	27.50	431.40
Remote volunteer	173.33	175.63	13.84	183.32

Regarding the evaluation of the **round-trip time (RTT)**, to cope with the high volume of MQTT messages exchanged between smart boxes and gateway while measuring the RTT, a specific methodology with negligible impact on communication has been implemented. As described in Section 3.2.4, the RTT is recorded every 10 seconds for a different sensor message, covering a total of six sensors: temperature, respiratory rate, heart rate, oxygen saturation, IMU, and ECG. Therefore, the RTT for each individual sensor is extracted approximately every 60 seconds.

**Fig. 17:**
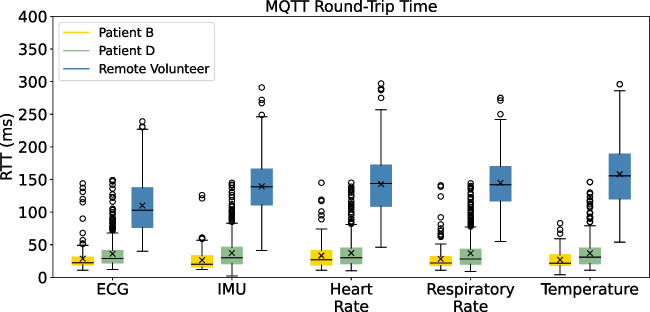
MQTT round-trip-time (RTT) by sensor data, between smart boxes and gateway

Figure 17 provides an insight into the round-trip time measurements between the smart boxes and the gateway, presented by each sensor over the three-day period of the pilots. As with previous results, the boxplots display results that are representative of the different participant mobility groups. Due to issues with oximeter malfunction and cases where certain patients chose not to use the oximeter, the oxygen saturation values were removed from the metrics, as described before. It can be seen that the RTT measurements for in-hospital patients are extremely low, as expected, at around 28 to 45 meters, since all devices are physically close to each other and on the same network. The round-trip times for patient D are longer than those of patient B, which can be explained by patient D’s higher mobility. Substantial difference can be seen when comparing with the results for the remote participant, who has much higher RTT values, between 90 and 180 meters, and a larger scatter. This is mainly due to the fact that the devices involved are connected to different networks, as in this case the smart box is connected to the test subject’s home network and the gateway was hosted on an external network. Table 6 provides additional statistical insight into the RTT performance of each participant, detailing the mean, median, standard deviation, and minimum and maximum RTT values for the MQTT communication between the smart boxes and the gateway. Upon analyzing the results, it becomes evident that the RTT is largely consistent among participants, with the exception of the remote volunteer who presents longer times.

**Table 6:** RTT performance in MQTT communication between smart boxes and gateway

	Round-trip time (ms)				
Participants	Mean	Median	Std Dev	Min	Max
Patient A	33.46	28.00	19.54	10.00	136.00
Patient B	28.71	23.00	18.87	4.00	145.00
Patient C	38.45	30.00	25.79	6.00	148.00
Patient D	36.94	29.00	23.74	2.00	149.00
Patient E	33.95	30.00	16.93	13.00	116.00
Patient F	38.17	34.00	21.27	14.00	147.00
Patient G	41.76	33.00	29.81	2.00	149.00
Patient H	39.92	29.00	29.90	9.00	149.00
Patient I	45.22	30.00	35.97	2.00	148.00
Remote volunteer	138.73	136.00	49.19	40.00	297.00

### FHIR-based Wi-Fi communication between gateway and HIS

Each time an EHR is sent from the gateway to the HIS for a particular sensor, the **RTT** is measured and logged. During the pilot, HIS sensor data requests were set to a 30-min period. ECG and IMU results are excluded from this analysis for specific reasons. In the case of the ECG signal, the interpretation of the data consists of compiling a chart composed of multiple readings, rather than evaluating individual ECG readings. For the IMU sensor, continuous data transmission and acquisition would also be required to collect meaningful data that allows conclusions to be drawn about the patient’s posture. As such, Glintt’s Globalcare HIS solution does not yet support displaying ECG and IMU data, and therefore the corresponding sensor data is not sent to the HIS.

**Fig. 18:**
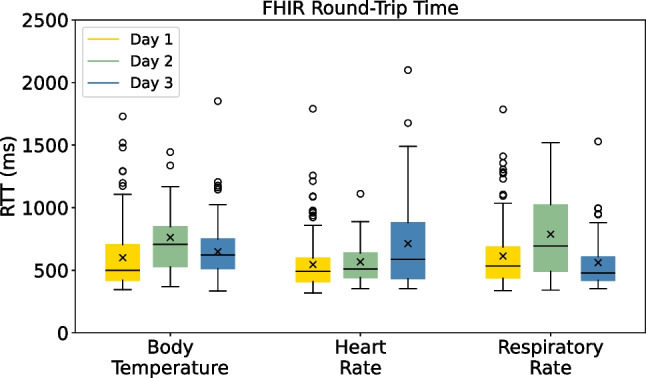
FHIR round-trip-time (RTT) between gateway and HIS according to sensor data

**Fig. 19:**
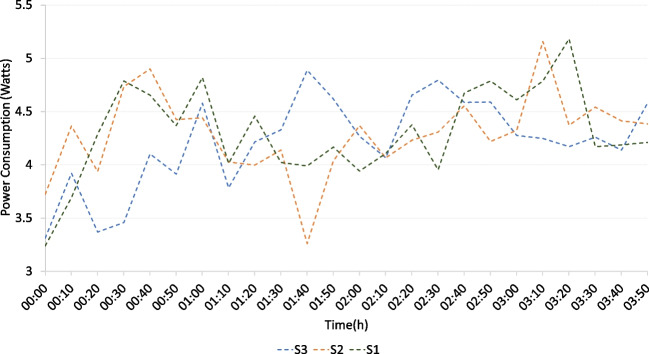
Power consumption of the smart boxes during the pilot

The results in Fig. 18 represent boxplots for the FHIR request round-trip time, which discriminate the RTT per sensor (body temperature, heart rate, and respiratory rate) for all patients per pilot day. These show that the RTT between the gateway and the HIS is generally stable and data collection is successful for all sensors involved. Since FHIR requests are transmitted over HTTP, which is inherently heavier than MQTT for instance, and since Glintt Healthcare Services’ solution is hosted in the cloud, it is understandable why we observe both higher values and greater variance in the data when we compare it to the RTT recorded between the smart boxes and the gateway. When looking at Fig. 18, fluctuations across the pilot days become apparent. These can be attributed to variations in network load and the intricacies of data processing within Glintt’s solution prior to transmitting the HTTP responses. However, if we assess the results achieved, we can confidently consider these deviations to be acceptable. This is primarily due to the fact that the frequency of data requests defined by healthcare professionals, which was set at a half-hourly interval, enabled seamless and efficient data exchange with extremely low load via this communication channel.

Accordingly, we have extracted the **throughput** for the FHIR-based Wi-Fi communication between the gateway and the HIS, taking into account each pilot day. On the first day, the throughput was measured at 61.101 bps. On the second day, it decreased to approximately 44.707 bps, and on the last day, it increased again to 67.889 bps. These results are justified by the fact that only two patients were monitored on the second day, while three patients were monitored simultaneously on the first and third days. As expected, these low values can be attributed to the practice of requesting data subscriptions only at half-hour intervals per smart box.

### Performance of individual modules

As soon as the battery is connected, the biosticker emits a light signal, indicating that it is ready to read and transmit data. It was observed that each biosticker took on average about 2 seconds to display this initialization signal, demonstrating a fast and efficient **calibration startup**. During the pilot test, compliance with the prescribed 24 hours of continuous use of the biosticker battery was also analyzed. To facilitate this assessment, the graphical user interface provides a comprehensive battery status log, allowing to monitor whether the battery is over-discharging or functioning optimally, as these factors can influence the study outcomes. No anomalies in the biosticker’s **battery performance** were detected. The participating patients were monitored from 5 to 24 hours, and no issues were found regarding the battery’s lifespan. Additionally, the biosticker battery was subjected to laboratory tests, which confirmed that the battery can operate autonomously for up to 48 hours without interruption.

**Fig. 20:**
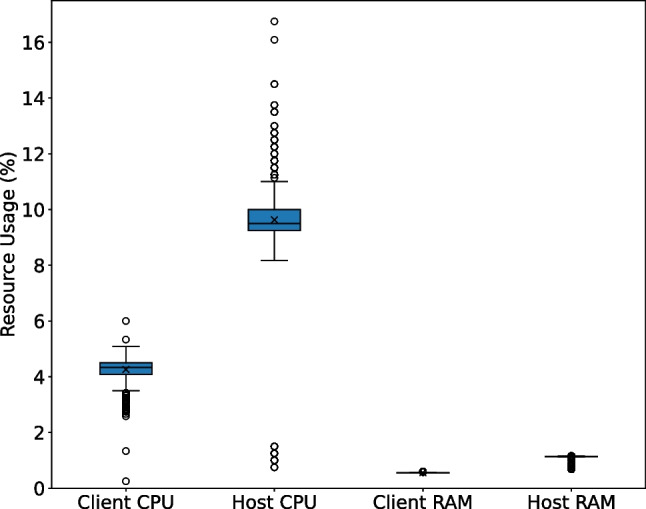
Average processor and memory usage of the GUI on the host and client device

Figure 19 illustrates **power consumption** of the smart box during the pilot. Data was recorded continuously and registered at 10-minute intervals for three smart boxes (S1, S2, and S3), resulting in values between 3.2 and 5.1 W. The observed peaks peak values correspond to the periods when the GUI deployed in the smart boxes experienced increased interaction and usage. These results indicate that the power consumption of a smart box is similar to that of a modern-day smartphone [44], exhibiting only a slight increase.

During the development stage, health professionals were consulted to ensure that the **WoW GUI** was intuitive and provided the essential functionalities, as described in Section 3.4.4. In the pilot, health professionals had the opportunity to interact with the GUI to analyze certain vital signs and fully explore all available features. No major issues were identified with the GUI menus and buttons, as all buttons and their functions complied with the previously defined requirements. The views of health professionals, as reflected in, the post-study questionnaires, are detailed in the next section.

**Fig. 21:**
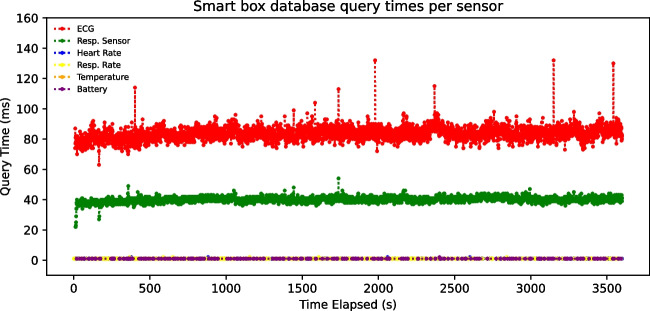
Measurements of query times over a period of 1 hour for data acquisition and GUI requests

Regarding the **computational cost** of the GUI, it proved to be lightweight on both the host (back-end) and the client (front-end), as shown in Fig. 20. On the smart box host, it consumed about 10% of CPU time and over 1% of memory. Considering that the host is a low-powered Raspberry Pi 4B device, we can conclude that the GUI is efficient. The resource consumption of the client front-end was even lower, namely at around 5% of the processor usage and less than 0.5% of the memory usage, on a standard laptop. The fact that the GUI is deployed as a web-based interface in a host-client paradigm is advantageous in this regard, as native applications usually have higher CPU and memory utilization.

**Fig. 22:**
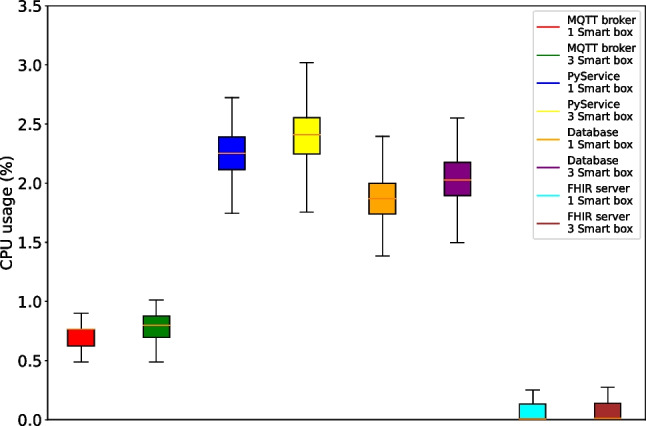
Average CPU usage per service in the gateway

Furthermore, the data acquisition script and the GUI must perform multiple queries per second to the local MongoDB database in the smart box to store the incoming data and display the data in the GUI. It is therefore to be expected that the query times will gradually increase during the operation of the system. However, as can be seen in Fig. 21, this is not the case. Query times remain stable and fast for all sensors (under 100 meters), with the ECG showing longer values compared to the other sensors due to the larger amount of data associated with each transaction.

**Fig. 23:**
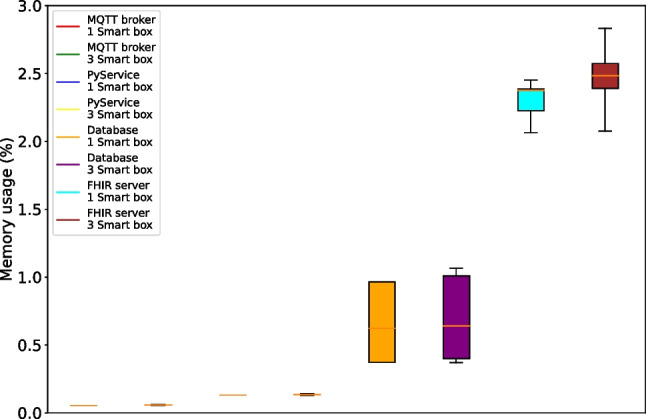
Average memory usage per service in the gateway

Lastly, the resource utilization by the services running in the gateway was also measured. The gateway is a central point of the system, responsible for the simultaneous processing and storage of data from all smart boxes and for interfacing with the HIS. As shown in Figs. 22 and 23, the increase in resource utilization from managing 1 to 3 smart boxes is not linear to the number of smart boxes. Instead, the CPU and memory utilization increases only minimally when smart boxes are added to the system. In terms of CPU usage, the data processing service PyService and the PostgreSQL database storage service are the most resource-intensive services at around 2% each. In terms of memory utilization, only the FHIR server shows meaningful usage at around 2.5%. These computational cost results show that the Intel NUC is extremely suitable to act as the central gateway in our system and can easily handle higher scaling up to dozens, if not hundreds, of patients. As with any IoT system, the observed low computational costs enable several benefits, including energy savings, cost reduction, improved performance, robustness and scalability, reduced latency, and even improved security.

**Fig. 24:**
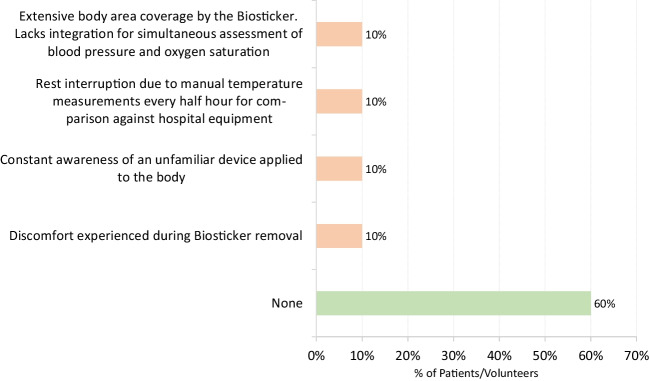
Post-study remarks shared by participants

**Fig. 25:**
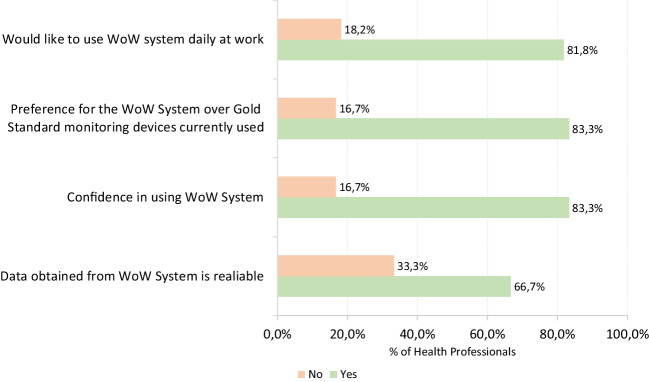
Post-study feedback from healthcare professionals on the WoW system

### System usability and user satisfaction questionnaires

After taking part in the pilot, ten surveys were completed by participants (9 patients and 1 volunteer), while 12 surveys were answered by health professionals. The health professionals who participated in the surveys included the nurses who applied the biostickers to the patients and others who were present in the application process during the 3 days of the study. Considering both patient and volunteer perspectives, the focus was on the comfort of using the biosticker during the monitoring period. Conversely, from the perspective of the healthcare professionals, the focus was on determining their perceptions of the proposed innovative system and highlighting its contrast with the standard and widely used monitoring techniques.

Participants were given the opportunity to share their remarks and concerns about the proposed system after conducting the study. As depicted in Fig. 24, 60% of participants did not experience any discomfort during the monitoring period. The main criticisms included slight discomfort when removing the biosticker patch, continuous awareness of an unfamiliar attached device, and the lack of simultaneous blood pressure and oxygen saturation measurement. One patient also reported intermittent fatigue due to the half-hourly recording of vital signs resulting from the pilot design. This led to rest disruptions as the nurse sought the patient’s permission for temperature measurements. Some patients also reported that the biostickers cover a large area of the body. Despite these findings and limitations, 78% of participants highlighted the wireless nature of the system as a significant strength. A further 22% emphasized the mobility that the system enables, as well as the ease and speed of application to the body.

Taking into consideration the survey of healthcare professionals, it was found that 66.7% of respondents believe that the data collected by the WoW system is reliable (see post-study feedback chart from healthcare professionals in Fig. 25). Furthermore, 83.3% of respondents expressed a preference for the WoW system over the monitoring devices currently used in the hospital and manifested a high level of confidence in using the system. Of the 12 respondents, 50% gave the highest score to the graphical user interface, citing its attractiveness, ease of use, and confidence-inspiring design. One respondent gave it a score of 9, four gave it a score of 8, and only one respondent gave it a score of 2. The main strength highlighted by healthcare professionals was the wireless nature of the system, allowing patients to have unrestricted mobility. However, weaknesses pointed out by health professionals included adhesion issues for patients with significant hairiness, the inability to measure blood pressure and oxygen saturation with a single equipment, and the challenge of performing patient hygiene without removing the electrical elements.

**Fig. 26:**
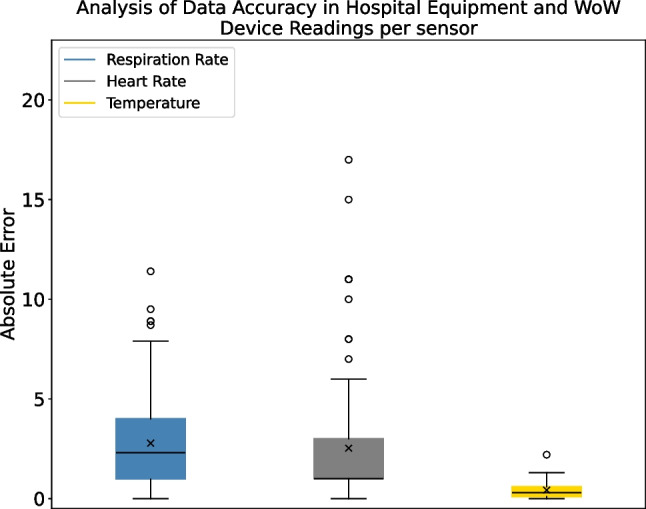
Accuracy of WoW vital signs. the gold standard heart rate data is determined by the Mindray iMEC15 patient monitoring system, the temperature by a digital thermometer, and the respiration rate by manual counting

### Comparison of WoW vital data against gold standard measurements

A statistical error analysis was carried out in which the data from gold standard measurements was compared with the data from the WoW sensors. The boxplot in Fig. 26 presents the absolute error in the measurement of respiratory rate, heart rate, and temperature, measured by the absolute difference between the gold standard values and our sensors. This analysis includes data collected over a 3-day period from all patients, except the remote volunteer. In addition, the analysis excludes the ECG signal, due to intermittent disruptions in the hospital equipment and malfunctioning electrodes.

According to the nurses, the most accurate method of measuring respiratory rate is manual counting by a trained professional; therefore, we considered this the gold standard. The respiration rate provided by the WoW system showed a mean difference of $2.88\pm 2.24$ units from the manual counting method. Although the perceived gap may seem considerable, we found a comparable, albeit slightly smaller difference of $2.38\pm 2.15$ units when compared to standard hospital patient monitoring devices, specifically the Mindray iMEC 15. Note that typical respiration rate values range between 12 and 25 breaths per minute.

The heart rate values compared with the aforementioned hospital device show an average deviation of 3 units. Typical heart rate values range between 68 and 95 beats per minute. A digital armpit thermometer was used to measure gold standard temperature. Patient temperatures ranged from 36 to 37 ^∘^C. The WoW temperature data presents a small deviation of approximately 0.4 ^∘^C between the biosticker temperature sensor and the hospital thermometer used.

Overall, the WoW system demonstrated an appropriate alignment with the current hospital monitoring technique. It is noteworthy that some of the recorded errors are due to different averaging times between the proposed system and the hospital equipment or to manual measurements. Besides the statistical analysis performed, a large-scale clinical validation would be required to fully characterize the precision and accuracy of the sensors.

## Conclusion

In this study, we deployed and validated a smart bed architecture in a real clinical setting where patients used wireless monitoring kits during their treatment in the hospital. We evaluated the reliability of communication using different protocols and specially designed performance metrics that included factors such as initialization times, dropped connections, packet loss, throughput, round-trip time (RTT), and received signal strength indicator (RSSI), among others. Additionally, individual performance indicators were evaluated, covering aspects such as the smart box’s power consumption, biosticker calibration, battery autonomy, and GUI testing. To further deepen the evaluation of the architecture, a comparative analysis was conducted between the WoW sensors and gold standard measurements in the hospital. For this purpose, we performed an error analysis on vital signs data recorded from patient monitoring at half-hourly intervals with both systems.

Feedback from patients participating in the pilot, and input from the healthcare professionals involved, was carefully considered to assess the current state of the system, identify its strengths, and highlight areas for future improvement. The results obtained confirm the reliability of communication between the different modules, with stable, efficient, and lightweight connections across all communication channels. Even in an uncontrolled network environment during remote testing, the system has consistently demonstrated its efficiency and stability at performing the required tasks. This opens up new possibilities for a wider adoption of wireless monitoring systems for untethered patients in the future.

## Future work

Following the pilot study, there are still challenges and improvements that we consider opportune to address, such as follows:

- Improve patient skin adhesion by exploring ergonomic and effective solutions for biosticker application and removal that alleviate the challenges associated with excessive hairiness or reduced skin elasticity.
- Integrate the monitoring of blood pressure and oxygen level by exploring methods to measure these parameters without requiring additional equipment to further improve the user experience and completeness of the system.
- Adjustments to the biosticker’s design, including its closure and soft-printed electronics, protecting the battery and enhancing the reliability and durability of components such as the respiratory sensors, making them less susceptible to interference or data corruption from actively mobile patients.
- Strengthen BLE connectivity, by migrating the current Mbed Os implementation to Zephyr OS, which natively supports LE Secure Connections pairing to further protect data acquisition, and improve connection stability and reliability (see [45]).
- Include additional features to the user interface, such as support for different display languages and a local authentication mechanism to promote both security and inclusivity for all patients.
- Enhance device setup and update procedures by creating automated routines for device provisioning and controlled software updates, simplifying the setup process for users without elaborate system knowledge.
- Integrate machine learning classification methods for prediction based on sensor fusion and historical patient data to obtain automatic categorization of patient status and detect critical conditions (e.g., imminent heart attack, falls) in time to alert the healthcare professionals.
- In the course of the previous point, as number of users, and computing and storage requirements increase, the system would benefit from transferring relevant tasks such as data fusion, analysis, storage and transmission to edge or cloud servers to promote scalability.

Despite these open challenges, the system has proven itself as an innovative and efficient solution, that has been well received by both the healthcare community and the patients, and offers promising prospects for future implementations of remote, wireless, non-intrusive, convenient, and seamless untethered patient monitoring.

## Data Availability

The data that support the findings of this study are available from the first author upon reasonable request.

## Footnotes

1 https://inovglintt.com/financiamento/wow/

2 Patients/Volunteers Feedback Survey: https://forms.gle/jMHQoTnPnLhnL22b9 Health Professionals Feedback Survey: https://forms.gle/pEwpSY3YDwkM7m6t8
